# Genome assembly of two diploid and one auto-tetraploid *Cyclocarya paliurus* genomes

**DOI:** 10.1038/s41597-023-02402-w

**Published:** 2023-08-02

**Authors:** Yinquan Qu, Xulan Shang, Shengzuo Fang, Xingtan Zhang, Xiangxiang Fu

**Affiliations:** 1 Nanjing Forestry University, Co-Innovation Center for Sustainable Forestry in Southern China, Nanjing, 210037 China; 2 Fishery College, Zhejiang Ocean University, Zhoushan, Zhejiang 316022 China; 3 Shenzhen Branch, Guangdong Laboratory of Lingnan Modern Agriculture, Genome Analysis Laboratory of the Ministry of Agriculture and Rural Affairs, Agricultural Genomics Institute at Shenzhen, Chinese Academy of Agricultural Sciences, Shenzhen, 518120 China

**Keywords:** Genome assembly algorithms, Genome evolution, Structural variation

## Abstract

*Cyclocarya paliurus*, an endemic species in the genus Juglandaceae with the character of heterodichogamy, is one of triterpene-rich medicinal plants in China. To uncover the genetic mechanisms behind the special characteristics, we sequenced the genomes of two diploid (protandry, PA-dip and protogyny, PG-dip) and one auto-tetraploid (PA-tetra) *C. paliurus* genomes. Based on 134.9 (~225x), 75.5 (~125x) and 271.8 Gb (~226x) subreads of PacBio platform sequencing data, we assembled 586.62 Mb (contig N50 = 1.9 Mb), 583.45 Mb (contig N50 = 1.4 Mb), and 2.38 Gb (contig N50 = 430.9 kb) for PA-dip, PG-dip and PA-tetra genome, respectively. Furthermore, 543.53, 553.87, and 2168.65 Mb in PA-dip, PG-dip, and PA-tetra, were respectively anchored to 16, 16, and 64 pseudo-chromosomes using over 65.4 Gb (~109x), 68 Gb (~113x), and 264 (~220x) Hi-C sequencing data. Annotation of PA-dip, PG-dip, and PA-tetra genome assembly identified 34,699, 35,221, and 34,633 protein-coding genes (90,752 gene models) or allele-defined genes, respectively. In addition, 45 accessions from nine locations were re-sequenced, and more than 10 × coverage reads were generated.

## Background & Summary

*Cyclocarya paliurus* (Batal.) Iljinskaja (wheel wingnut) is an important medicinal woody plant, which widely distribute in mountanous areas of south and southeast China^1^. Its leaves are often used in traditional Chinese medicine to treat hypertension and diabetes due to its multiple bioactive compounds triterpenoids, especially species-specific triterpenoids, e.g. cyclocaric acid B (CA-B). However, evolutionary history and functions of triterpenoid-biosynthetic-related genes remain unknown. Moreover, the character of heterodichogamy with two temporally complementary genetic morphs in *C. paliurus*, significantly affects seed filling index and quality. However, the published *C. paliurus* genome^2^ with lower quality can’t support to expound above biological issues, further imped its widely application, such as metabolite yields and medicinal value. Our survey found that most of natural populations of *C. paliurus* are auto-tetraploid (2n = 4x = 64) with a base chromosome number of 16. However, populations distributed in areas along with 30° N in China possess diploid (2n = 2x = 32) individuals^3^. The *C. paliurus* genome, especially auto-tetraploid is characterized by great heterozygosity (1.97%), due likely to the fact that *C. paliurus*is is a outcrossing species.

Hence, we present chromosome-scale genome assemblies for auto-tetraploid (PA-tetra), and two diploid (PA-dip and PG-dip) *C. paliurus* that represent two flower morphs, protandry (PA) and protogyny (PG). We extracted total DNA from *C. paliurus* tissues, sequenced in Illumina Novaseq. 6000 with 150-bp paired-end (PE) reads length. After data filtering and preprocessing, 106.7, 86, and 291.7 Gb raw data were generated for PA-dip, PG-dip, and PA-tetra *C. paliurus*, respectively. We also sequenced constructed libraries using a PacBio Sequel II with P6-C4 chemistry, finally, 134.9, 75.5, and 271.8 Gb raw data were generated for PA-dip, PG-dip, and PA-tetra *C. paliurus*, respectively. In addition, High-throughput Chromatin Conformation Capture (Hi-C) sequencing was used for the chromosomal level assemblies. A total of 196, 190, and 687 million of 150-bp paired-end reads were produced on Illumina Novaseq. 6000 system. The final genome assembly size in PA-dip, PG-dip, and PA-tetra *C. paliurus* were 586.62 Mb, 583.45 Mb, and 2.38 Gb, containing 1,101 contigs (N50 = 1.9 Mb), 921 contigs (N50 = 1.4 Mb), and 9,744 contigs (N50 = 430.9 kb), respectively. Structural annotation of three genomes yielded 34,699 (PA-dip), 35,221 (PG-dip), and 34,633 (PA-tetra) protein-coding genes, and no less than 94.4% of highly conserved embryophyta genes completely presented in the genome assembly. To explore the population structure and evolutionary history of *C. paliurus* population, More than 10 × coverage of per sample for 35 tetraploid and 10 diploid *C. paliurus* growing in nine locations were generated from 150-bp Illumina short reads, respectively. A total of 3.89 and 26.67 million variants were respectively identified from diploid and tetraploid populations.

These three chromosome-level genomes of *C. paliurus* will greatly facilitate researchers to uncover the genetic mechanisms behind the special characteristics of *C. paliurus* species, including heterodichogamy, the origination and polyploidization, and the pathway of triterpenoids biosynthesis. In addition, the genome assembled in this study provides a valuable genomics resources for future efforts to protect the vulnerable *C. paliurus* and identify key functional genes.

## Methods

### Sample collection, library construction and sequencing

**Fig. 1:**
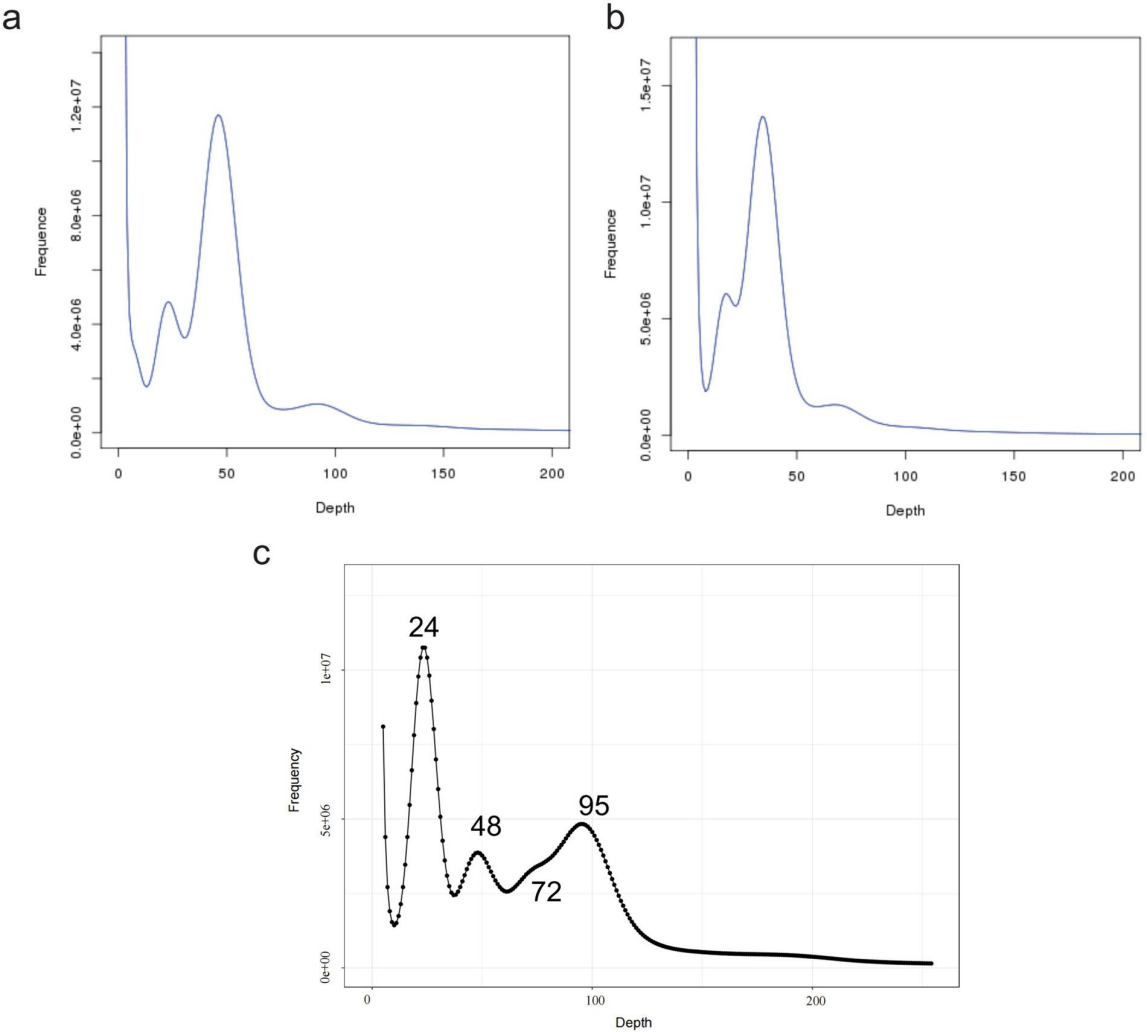
K-mer (21-mer) distribution and estimation of genome size of *C. paliurus*. (**a**) PA-dip. (**b**) PG-dip. (**c**) PA-tetra.

Leaves of two diploid *C. paliurus* (PG-dip and PA-dip) and one auto-tetraploid (PA-tetra) for genome sequencing were collected from plants grown in germplasm bank of *C. paliurus*, which located in Baima experimental field, Nanjing, Jiangsu province, China. After collecting, tissues were immediately frozen in liquid nitrogen and stored at −80 °C. Genomic DNA was isolated and extracted using the QIAGEN DNaesy Plant Mini Kit and sequenced in Illumina Novaseq. 6000 with 150-bp paired-end (PE) reads length. A total of 106.7, 86, and 291.7 Gb subreads were generated, comprising ~178× , 143× , and 243× coverage of the estimated genome sizes on the Illumina platform (Table 1 and Fig. 1). In addition, high-molecular-weight genomic DNA was sheared to a ~20 kb targeted size, followed by damage repair and end repair, blunt-end adaptor ligation and size selection with a Blue Pippin system. The final libraries were subsequently sequenced using a PacBio Sequel II with P6-C4 chemistry. After data filtering and preprocessing, 134.9, 75.5, and 271.8 Gb raw data were generated for PA-dip, PG-dip, and PA-tetra *C. paliurus*, respectively. Hi-C libraries were created from tender leaves of *C. paliurus* seedlings as described^4^. Briefly, the leaves were fixed with lysed, formaldehyde, and then the cross-linked DNA digested with HindIII over-night. Sticky blunt ends were biotinylated and proximity-ligated to form chimeric junctions, and then physically sheared to a size of 500–700 bp. Chimeric fragments representing the original cross-linked long-distance physical interactions were then processed into paired-end sequencing libraries. A total of 65.4, 68, and 264 Gb reads were produced for PA-dip, PG-dip, and PA-tetra *C. paliurus*, respectively on Illumina Novaseq. 6000 system, comprising ~106× , 113× , and 220× coverage of the estimated genome sizes. Hi-C sequencing was assessed using HiC-Pro^5^ program and the results showed a decent proportion of validate reads (no less than 80% in three *C. paliurus* Hi-C libraries), suggesting high quality of the Hi-C data (Table 1).

**Table 1:** Sequencing information for the assemblies.

Library type	Insert size	Raw data (Gb)			Coverage (×)		
		PA-dip	PG-dip	PA-tetra	PA-dip	PG-dip	PA-tetra
Illumina Paired-end	200 bp	106.7	86	291.7	178	143	243
PacBio Sequel II	20 kb	134.9	75.5	271.8	225	125	226
Hi-C	200 bp	65.4	68	264	109	113	220
Total	—	307	229.5	827.5	512	381	689

The stems, leaves, male floral buds, and female floral buds in various PA-dip, PG-dip, and PA-tetra individuals were respectively collected in May. At this stage, all the flower buds began to protrude. Three biological replicates of the extracted RNA were sequenced on Illumina Novaseq platform, and 6 Gb RNA-seq raw data for each sample was generated. Total RNA was isolated from each tissue using E.Z.N.A Plant RNA Kit (Omega Bio-tek, Doraville, GA), and then purified with RNase-Free DNase I (Takara Company, China). 1% agarose gel was used to evaluate the RNA contamination and degradation. The purity was further monitored using ultraviolet spectrophotometer (Implen, München, Germany). Samples with RIN values higher than 8 were used for downstream cDNA library preparation. The cDNA libraries construction was performed with NEBNext® UltraTM RNA Library Prep Kit according to the manufacturer’s instruction. The Agilent Bioanalyzer 2100 system was performed for library quality assessing and short paired-end reads were generated from the libraries preparations based on Illumina Novaseq sequencing platform.

### Genome size estimation

To estimate the genome size for three *C. paliurus*, Illumina short reads were recruited to determine the distribution of *K*-mer values using published Estimate_genome_size.pl scripts (https://bioinformatics.uconn.edu/genome-size-estimation-tutorial/). The genome size was estimated using the following formula: the number of *K*-mers divided by the depth of peaks (*K*-mer-number/depth). The size of the haploid genomes for PA-dip and PG-dip *C. paliurus* were estimated to be approximately 538.16 Mb and 574.97 Mb (Fig. 1), which are similar with the genome size of *Pterocarya stenoptera*^6^, the species in the same family with *C. paliurus*. In addition, the estimated genome size of PA-tetra *C. paliurus* is 2065.41 Mb and is likely a high heterozygosity plant (Fig. 1).

### Preprocessing and genome assembly

All sequencing subreads generated from Pacbio SMRT Sequencer were self-corrected and trimming using CANU (v.1.7)^7^ with optimized parameters (corOutCoverage = 100). Then, the overlaps distributed in all preassembled error-corrected reads were identified using FALCON (https://pb-falcon.readthedocs.io/en/latest/). The overlaps were applied to construct a directed string graph following the Myers’ algorithm. Contigs were constructed by finding the paths from the string graph (falcon_sense_option = --output_multi --min_idt 0.70 --min_cov 4 --max_n_read 300 --n_core 22; overlap_filtering_setting = --max_diff 200 --max_cov 200 --min_cov 2--n_core 30). To correct the systematic errors of PacBio platform sequencing, the Illumina short reads from the same three *C. paliurus* individuals were mapped against CANU initial genome assembly using BWA^8^ mem. The variants considered as sequencing errors were polished using Pilon pipeline^9^.

The quality of paired-end Hi-C reads were firstly evaluated using HiC-Pro^5^, and the Hi-C data was aligned to the contig-level assembly using BWA. After data analyzed by juicer^10^, the mis-joined and long contigs were interrupted using the 3D-DNA pipeline^4^, then iterative scaffolding was carried out following the several rounds of error correction from 3D-DNA. Properly paired reads chosen for the reassembly, should satisfy the condition that both paired ends are properly aligned according to the aligner. The contigs corrected by Hi-C interactions were successfully linked into 16 pseudo-chromosomes in PG-dip and PA-dip, and 64 pseudo-chromosomes with four sets of monoploid chromosomes in PA-tetra *C. paliurus* using the ALLHiC pipeline^11^.

### Repeat annotation

Repetitive sequences in three *C. paliurus* genomes were identified using same pipeline. Firstly, RepeatModeler (http://www.repeatmasker.org/RepeatModeler/) and RepeatMasker (http://www.repeatmasker.org/) were used to *de novo* predict and annotate the constructed transposable elements (TEs). Secondly, TEclass (v2.1.3)^12^ was performed to categorize and classify the unknown TEs. Thirdly, Tandem Repeat Finder (TRF) (v4.07)^13^ and LTR_Finder (v1.05)^14^ were used to detect intact long terminal repeat (LTR) retrotransposons and tandem repeat, respectively. Finally, two pipelines LTRharvest (v1.5.10)^15^ and LTR_retriever^16^ were used to construct a high-quality LTR library. In total, the PA-dip, PG-dip, and PA-tetra *C. paliurus* genomes were found to contain 282.25 Mb, 316.95 Mb, and 1,154.35 Mb repetitive sequences, accounting for 48.1%, 54.3%, and 48.4% of their length, respectively (Table 2). As shown in Table 2, the most common classifications assigned to these repetitive fractions were retroelements, ranging from 35.7 to 37.3% in the three genomes.

**Table 2:** Repeat annotation of the three *C. paliurus* genomes.

			Repeat Length (Mb)			Percent of assembled genome (%)		
			PA-dip	PG-dip	PA-tetra	PA-dip	PG-dip	PA-tetra
Total repeat fraction			282.25	316.95	1,154.35	48.1	54.32	48.41
Class I: Retroelement			211.85	217.88	851.72	36.1	37.34	35.72
	LTR Retrotransposon		115.38	119.2	451.36	19.66	20.43	18.93
		Ty1/Copia	31.46	34.29	123.3	5.36	5.88	5.17
		Ty3/Gypsy	36.63	37.25	124.15	6.24	6.38	5.21
		Other	47.29	47.67	203.91	8.06	8.17	8.55
	non-LTR Retrotransposon		76.21	77.89	312.45	12.99	13.35	13.1
		LINE	71.87	73.46	289.88	12.25	12.59	12.16
		SINE	4.34	4.43	22.57	0.74	0.76	0.95
	Unclassified retroelement		20.26	20.79	87.91	3.45	3.56	3.69
Class II: DNA Transposon			61.25	64.39	257.05	10.44	11.03	10.78
	CMC		10.36	8.76	36.43	1.76	1.5	1.53
	hAT		11.42	13.28	45.15	1.95	2.28	1.89
	Mutator		2.47	2.07	8.79	0.42	0.35	0.37
	PIF/Harbinger		2.67	1.61	12.11	0.45	0.28	0.51
	Other		34.34	38.67	154.57	5.85	6.63	6.48
	Helitron		1.59	0.89	4.22	0.27	0.15	0.18
Tandem Repeats			13.87	12.9	72.59	2.36	2.21	3.04
Unkown			9.4	10.11	40.05	1.6	1.73	1.68

### Gene annotation

To obtain high quality protein-coding genes for three *C. paliurus* genomes, we employed a comprehensive strategy from ab initio gene prediction, RNA-seq-based prediction, and homology-based prediction. RNA-seq-based prediction was performed with the Trinity^17^ software, which was used to predict protein-coding genes based on RNA-seq data from four different tissues (stems, female floral buds, male floral buds, and leaf buds) of *C. paliurus*, genome-guided assembly and *de novo* assembly. RSEM^18^ was used to quantify the fragments per kilobase per million (FPKM) expression level of assembled transcripts, and low quality transcripts were filtered with default criterion. The comprehensive transcript library from the filtered transcripts were constructed by PASA^19^ program. Then, the filtered transcripts screened from PASA were aligned to protein sequences of six species (*Oryza sativa*, *Vitis vinifera*, *Carica papaya*, *Morus notablis*, *Solanum tuberosum* and *Arabidopsis thaliana*). For protein-coding genes annotation, MAKER2^20^ pipeline run by two rounds. For the first round, MAKER2 was employed to generate consensus genes by integrating three annotation strategies (SNAP^21^, GENEMARK^22^ and AUGUSTUS^23^). After that, the MAKER2 was used to integrate multiple tiers of coding evidence, including ab initio gene prediction, transcript and protein evidence and generate a comprehensive set of protein-coding genes. After the first round, the predicated gene models with annotation edit distance (AED) values < 0.2 were selected for model re-training. Finally, the gene annotation improvement was generated by the second round of MAKER2. After filtering, a total of 34,699, 35,221, and 90,752 gene models were annotated in PA-dip, PG-dip and PA-tetra *C. paliurus*, respectively.

### Samples re-sequencing and variant calling

In 2019 and 2020, we visited nine populations of *C. paliurus* distributed across south of China, and sampled 45 wild individuals including 10 diploid and 35 tetraploid accessions (Table 3). In addition, one walnut species (*Juglans regia*) was performed as an outgroup. All samples containing diploid and tetraploid *C. paliurus* were sequenced on Illumina Novaseq. 6000, and generated the expected more than 10 × coverage short reads (150 bp paired-end). Clean data was obtained by trimming adapters and low-quality reads (Q < 30) using Trimmomatic (v0.32)^24^, followed by aligning against the reference genome of PA-dip *C. paliurus* by BWA. The variant calling was carried out by GATK (v4.0.3.0)^25^. Firstly, GATK HaplotypeCaller was performed to identify general variants from each individual, and a single variant calling file was generated by GenotypeGVCFs software. This two-step approach was carried out to ensure variant accuracy, which included re-genotyping and quality re-calibration in the combined vcf file.

**Table 3:** *C. paliurus* and outgroup (*Juglans regia*) species used for the population genomics analysis.

Location	ID	Altitude(m)	Latitude (°E)	Longitude (°N)	Ploidy	X-mean	Reference	Coverage (×)
Hefeng County, Hubei, China	A1	1,516	110.456345	29.955051	Diploid	10,169	19,009	21.83
	A2	1,340	110.460312	29.971876	Diploid	10,171	19,270	10.33
	A3	1,494	110.462555	29.965052	Diploid	10,139	18,670	11
	A4	970	110.433548	29.855425	Auto-tetraploid	20,779	11,417	15.67
	A5	1,340	110.46006	29.971657	Diploid	10,945	19,855	11
Houhe National Nature Reserve, Hubei, China	B1	1,600	110.543877	30.069838	Diploid	9,890	18,292	10.33
	B2	1,629	110.544258	30.064539	Diploid	11,139	19,871	11
	B3	1,650	110.567062	30.066883	Diploid	10,413	18,835	10.83
	B4	1,630	110.600578	30.111212	Diploid	10,726	19,360	10.83
	B5	1,400	110.541718	30.085014	Auto-tetraploid	21,019	11,422	10.5
Huangshan, Anhui, China	C1	711	118.165787	30.104895	Auto-tetraploid	18,751	10,308	10.58
	C2	716	118.166092	30.105141	Auto-tetraploid	19,334	10,037	15.5
	C3	630	118.171951	30.100531	Auto-tetraploid	19,285	10,349	10.42
	C4	967	118.16922	30.159826	Auto-tetraploid	19,132	10,562	15.5
	C5	1,316	118.167465	30.152409	Diploid	9,865	17,568	21
Qingliangfeng Nature Reserve, Anhui, China	D1	680	118.893478	30.149855	Auto-tetraploid	19,005	10,207	15.33
	D2	907	118.893867	30.139042	Auto-tetraploid	18,595	9,907	10.92
	D3	685	118.894279	30.14976	Auto-tetraploid	20,008	10,466	15.17
	D4	674	118.856438	30.151596	Auto-tetraploid	18,590	10,122	15.17
	D5	1,406	118.842934	30.127003	Diploid	8,920	15,833	10.33
Chiu-ling Mountains, Jiangxi, China	E1	771	115.324646	29.091516	Auto-tetraploid	17,192	8,858	19.92
	E2	780	115.264275	29.015097	Auto-tetraploid	18,105	9,951	10.58
	E3	771	110.442207	29.965858	Auto-tetraploid	19,122	10,165	10
	E4	577	115.22229	29.029156	Auto-tetraploid	19,629	10,867	10.17
	E5	580	115.223457	29.033163	Auto-tetraploid	19,819	11,574	11.08
Tonggu County, Jiangxi, China	F1	875	114.219116	28.519285	Auto-tetraploid	19,905	11,494	11.25
	F2	781	114.206985	28.530428	Auto-tetraploid	19,391	10,981	10.83
	F3	793	114.20776	28.539653	Auto-tetraploid	19,336	10,509	11.33
	F4	812	114.220161	28.516615	Auto-tetraploid	18,581	10,251	11.67
	F5	762	114.20887	28.530558	Auto-tetraploid	18,945	10,298	11.25
Shucheng County, Anhui, China	G1	798	116.540108	31.037395	Auto-tetraploid	20,020	11,302	11.33
	G2	831	116.541092	31.037144	Auto-tetraploid	18,987	10,369	10.83
	G3	744	116.539253	31.039656	Auto-tetraploid	20,421	11,645	10.75
	G4	764	116.539048	31.039019	Auto-tetraploid	19,074	10,917	11.42
	G5	766	116.539162	31.037907	Auto-tetraploid	19,636	10,223	10.5
Jinzhongshan Nature Reserve, Guangxi, China	H1	1,810	104.950639	24.61725	Auto-tetraploid	20,681	10,922	15.08
	H2	1,829	104.952258	24.617875	Auto-tetraploid	21,125	11,951	11
	H3	1,819	104.954131	24.617858	Auto-tetraploid	21,361	10,270	10.75
	H4	1,698	104.953714	24.616278	Auto-tetraploid	20,620	10,870	11
	H5	1,593	104.950094	24.612328	Auto-tetraploid	20,276	11,120	10.83
Muchuan County, Sichuan, China	I1	1,217	103.791064	24.966956	Auto-tetraploid	20,636	10,749	19.92
	I2	1230	103.792557	28.965565	Auto-tetraploid	21,076	11,385	10.92
	I3	1,228	103.791656	28.965809	Auto-tetraploid	19,762	10,718	10.58
	I4	1192	103.793526	28.969936	Auto-tetraploid	18,226	9,970	10.75
	I5	1,200	103.791069	28.966961	Auto-tetraploid	18,658	9,868	11.25
Mei, haanxi, China	Outgroup	—	107.75	33.82	Diploid	—	—	23.1

X-mean represents the fluorescence intensity of the sample peak which is proportional to the DNA content of the cells. Reference mean of internal reference (diploid or tetraploid *C. paliurus*) for flow cytometry analysis. The data from Outgroup was referred to Zhang’s report^30^.

## Data Records

The whole genome sequencing raw data including Illumina short reads, PacBio long reads, Hi-C interaction reads, and re-sequencing data (SRP421615^26^) have been submitted to the NCBI Sequence Read Archive (SRA) database under BioProject accession number PRJNA931978. The assembled genome was deposited in GenBank under the accession number of GCA_029856945.1^27^, GCA_029856935.1^28^, and GCA_029856905.1^29^, respectively. The VCF files have been submitted to appropriate public Figshare database^30^.

## Technical Validation

### Quality assessment of the genome assembly

**Fig. 2:**
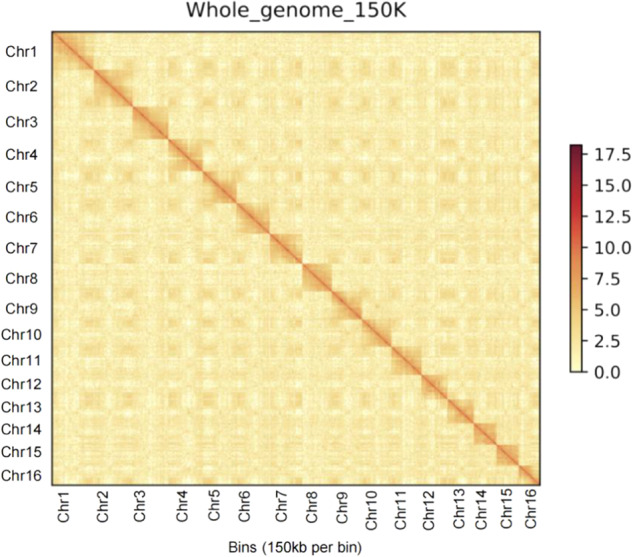
Genome-wide analysis of chromatin interactions at 150-kb resolution in PA-dip genome. The colored bar on the right represents the strength of interaction.

**Fig. 3:**
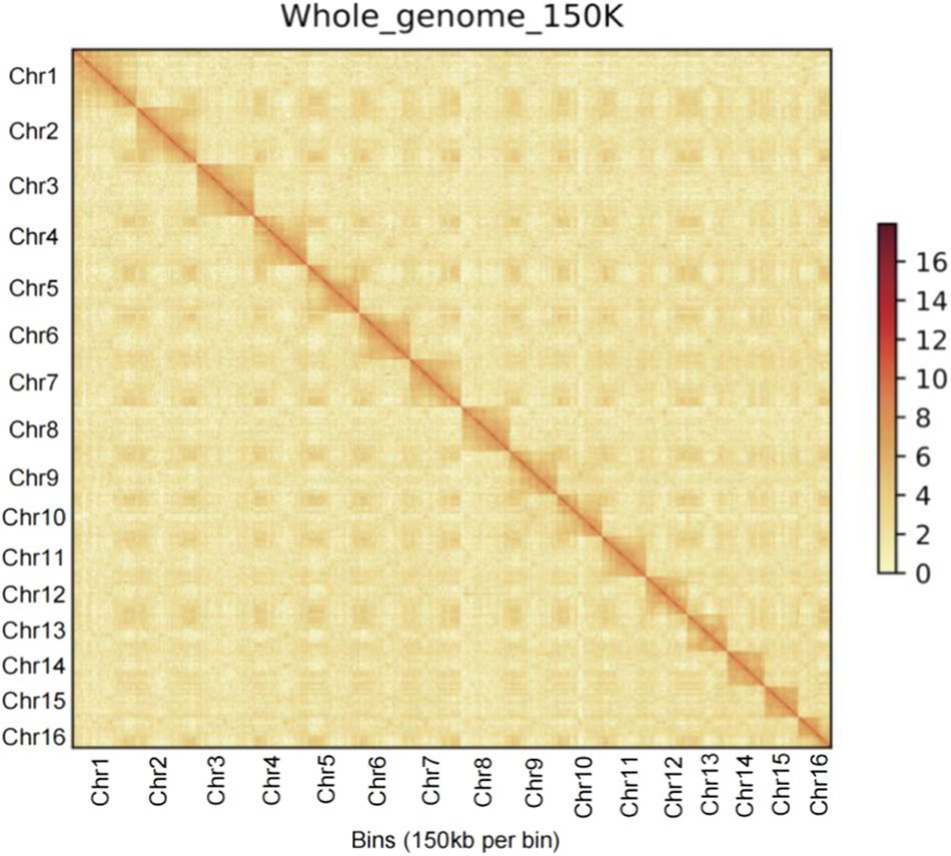
Genome-wide analysis of chromatin interactions at 150-kb resolution in PG-dip genome.

**Fig. 4:**
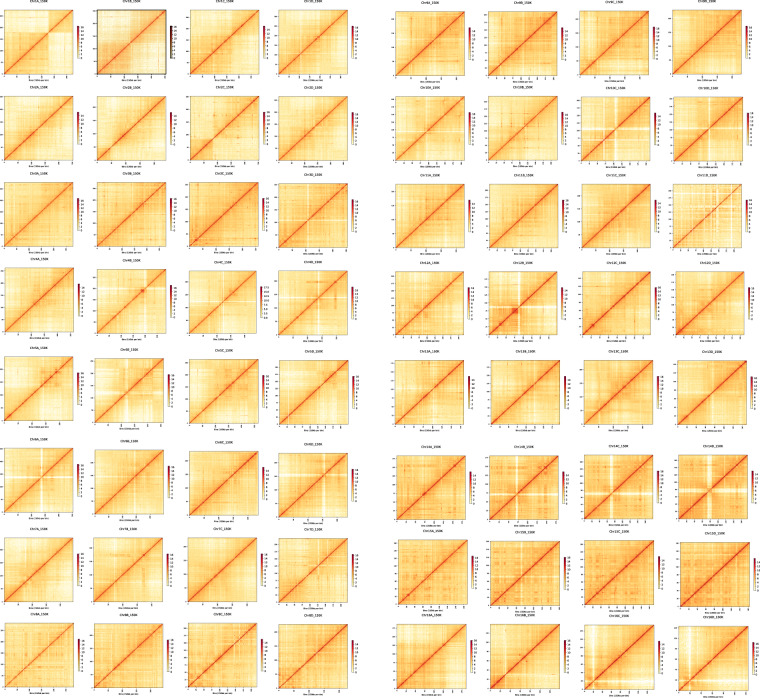
Genome-wide analysis of chromatin interactions at 150-kb resolution in PA-tetra genome.

The assemblies presented here are the two diploid (two mating types with PA and PG), and one haplotype-resolved assembly of auto-tetraploid *C. paliurus* genomes. The contig N50 for PA-dip, PG-dip, and PA-tetra *C. paliurus* were 1.93 Mb, 1.39 Mb, and 430.92 kb, respectively. Besides, 543.53 (92.65%), 553.87 (94.93%), and 2168.65 Mb (91.08%) of PA-dip, PG-dip, and PA-tetra, were respectively anchored to 16, 16, and 64 pseudo-chromosomes (Table 4). For completeness assessment of PA-dip, PG-dip, and PA-tetra *C. paliurus* genomes, Illumina short reads were mapped to the assembly genome. The global mapping ratio and properly paired ratio reached at least 98.89% and 93.72%, respectively (Table 5). Further, we also assessed the completeness of the genome assembly with the BUSCO version 3 based on embryophyta_odb10 (https://busco.ezlab.org/list_of_lineages.html). More than 95.2% of complete BUSCOs and less than 3.9% of missing BUSCOs were identified in assembly (Table 6). Finally, we evaluated the assembly of the 16 chromosomes for two diploid genomes and 64 chromosomes for tetraploid genome (Figs. 2–4). The anchored genome was split into bins of 150 kb in length. The number of Hi-C read pairs covered by any two bins was used to define the signal for the interaction between those bins, and these signal intensities were plotted in the form of a heat map. The signal intensities clearly divided the bins into 16 distinct groups for both two diploid genomes and concentrated on diagonal, demonstrating the high consistency of genome structure and quality.

**Table 4:** Chromosomal level genome assemblies.

ChrID	PA-dip (Mb)	PG-dip (Mb)	PA-tetra (Mb)			
			Hap A	Hap B	Hap C	Hap D
Chr1	46.29	46.58	41.27	38.01	42.45	50.08
Chr2	43.74	44.4	43.92	43.74	32.86	50.4
Chr3	39.6	41.58	41.77	41.49	38.96	38.16
Chr4	38.01	38.67	39.44	42.99	36.33	35.67
Chr5	37.99	37.97	37.28	39.01	39.72	33.65
Chr6	36.49	37.3	37.88	37.41	35.25	35.28
Chr7	36.56	37.08	44.92	35.67	40.88	25.69
Chr8	32.7	35.15	42.35	36.54	32.56	34.12
Chr9	33.84	34.68	33.98	31.55	34.36	34.52
Chr10	32.37	32.69	31.48	32.99	31.72	30.89
Chr11	33.71	32.21	33.96	38.48	36.88	23.04
Chr12	29	30.21	32.31	29.82	23.49	31.52
Chr13	28.95	29.25	27.51	29.46	35.23	22.26
Chr14	25.93	27.22	27.58	27.05	26.89	26.16
Chr15	24.52	24.85	25.3	24.02	25.61	24.33
Chr16	23.82	24.03	27.91	20.92	23.21	18.46
Total length of contigs (Mb)	586.62	583.45	2380.95			
Total length of chromosome level assembly (Mb)	543.53	553.87	2168.65			
Anchor rate (%)	92.65	94.93	91.08

**Table 5:** Statistics of Illumina short reads remapped to the assemblies.

	Percentage		
	PA	PG	PA-tetra
Total reads	100.00%	100.00%	100.00%
Mapped reads	98.89%	99.22%	99.51%
Paired reads	98.88%	99.49%	99.77%
Properly paired reads	93.72%	96.21%	96.99%
Unmapped reads	0.34%	0.21%	0.09%

**Table 6:** BUSCO assessment of genome assemblies.

Description	PA-dip		PG-dip		PA-tetra	
	Number	Percentage(%)	Number	Percentage(%)	Number	Percentage(%)
Complete BUSCOs(C)	1308	95.2	1326	96.4	1312	95.5
Complete and single-copy BUSCOs(S)	1178	85.7	1194	86.8	181	13.2
Complete and duplicated BUSCOs(D)	130	9.5	132	9.6	1131	82.3
Fragmented BUSCOs(F)	14	1	9	0.7	8	0.6
Missing BUSCOs(M)	53	3.8	40	2.9	55	3.9
Total BUSCO groups searched	1375	100	1375	100	1375	100

### Gene annotation validation

Gene annotation in three genomes were performed using a combination of RNA-seq-based, homology-based, and ab initio gene prediction. To improve the quality of gene prediction, the transcripts were removed if FPKM less than 1, while the protein sequences with coverage greater than 95% were reserved as candidate sequences. After the first round, the predicated gene models with annotation edit distance (AED) values < 0.2 were selected for model re-training. BUSCO (version 3) analysis for PA-dip, PG-dip, and PA-tetra *C. paliurus* were used to evaluate the completeness of protein-coding annotation using embryophyta_odb10 (https://busco.ezlab.org/list_of_lineages.html), showing more than 94.4% of BUSCOs value (Table 7).

**Table 7:** BUSCO analysis of annotation completeness.

Description	PA-dip		PG-dip		PA-tetra	
	Number	Percentage (%)	Number	Percentage (%)	Number	Percentage (%)
Complete BUSCOs(C)	1,323	96.2	1,323	96.2	1,299	94.4
Complete and single-copy BUSCOs(S)	1,203	87.5	1,200	87.3	178	12.9
Complete and duplicated BUSCOs(D)	120	8.7	123	8.9	1,121	81.5
Fragmented BUSCOs(F)	25	1.8	30	2.2	18	1.3
Missing BUSCOs(M)	27	2	22	1.6	58	4.3
Total BUSCO groups searched	1,375	100	1,375	100	1,375	100

### Validation population structure variant

SNPs were further filtered according to the following parameters: (1) SNPs presented only in one of the two pipelines (SAMtools/BCFtools and GATK); (2) SNPs in repeat regions; (3) non-biallelic SNPs; (4) SNPs with missing rate more than 40%; (5) SNPs were removed if the distance with nearby variant sites less than 5 bp; (6) SNPs with read depth more than 1,000 or less than 5. A total of 3.89 and 26.67 million variants were respectively generated from diploid and tetraploid populations, containing 3.55 and 23.08 million SNPs, and 0.34 and 3.60 million indels. We also identified 3,845 and 38,899 variants in genic regions, including 3,753 and 23,764 synonymous, 5,503 and 35,241 nonsynonymous, respectively (Table 8).

**Table 8:** Summary of genetic variations in *C. paliurus* populations.

Category	Diploid	Tetraploid
**Sequence variants**		
Total	3,886,832	26,674,995
SNPs	3,545,162	23,076,276
Indels	341,670	3,598,719
**Variants with effects on genes**		
SNPs that introduce stop codons	145	1,068
SNPs that disrupt stop codons	19	114
SNPs that induce alternative splicing	472	3,237
Indels located in genic regions	3,845	38,899
Frameshift variants	314	2,088
Nonsynonymous variants	5,503	35,241
Synonymous variants	3,753	23,764

## Data Availability

The sofware versions, settings and parameters used are described below. **(1) BWA:** version 0.7.12-r1039, default parameters; **(2) SAMtools/BCFtools:** version 0.1.19, default parameters; **(3) Trinity:** trinityrnaseq-2.9.0, default parameters; **(4) RSEM:** default parameters; **(5) HiSAT2:** default parameters;**(6) GATK:** version 4.0.3.0, default parameters; **(7) HiC-Pro:** version 2.10.0, default parameters; **(8) CANU:** version 1.7, parameters: batOptions = -dg 3 -db 3 -dr 1 -ca 500 -cp 50; **(9) FALCON:** version 0.2.2, default parameters; **(10) Pilon:** version 1.2.3, parameters: --mindepth 4 --threads 6 --tracks --changes --fix bases --verbose; **(11) Juicer:** version 0.0.2, default parameters; **(12) 3D-DNA:** version 180922, default parameters; **(13) ALLHiC:** version 0.9.8, default parameters; **(14) RepeatModeler:** RepeatModeler-open-1.0.11, default parameters; **(15) RepeatMasker:** version 2.1, default parameters; **(16) TEclass:** librf_lvq_pak-3.1, default parameters; **(17) Tandem Repeat Finder (TRF**): version 4.07, default parameters; **(18) LTR_Finder:** version 1.05, default parameters; **(19) LTRharvest:** version 1.5.10, default parameters; **(20) LTR_retriever:** version 2.9.0, default parameters; **(21) PASA:** version 2.0.2, default parameters; **(22) MAKER2: (23) SNAP:** version 2006-07-28, default parameters; **(24) GENEMARK:** version 3.9, parameter used: -f gf3; **(25) AUGUSTUS:** version 2.5.5, parameters: --species = arabidopsis; **(26) StringTie:** version 2.2.1, default parameters; **(27) Trimmomatic:** version 0.32, default parameters; **(28) GATK:** version 4.0.3.0, default parameters.
